# Dopamine-Dependent Plasticity Is Heterogeneously Expressed by Presynaptic Calcium Activity across Individual Boutons of the *Drosophila* Mushroom Body

**DOI:** 10.1523/ENEURO.0275-23.2023

**Published:** 2023-10-26

**Authors:** Andrew M. Davidson, Shivam Kaushik, Toshihide Hige

**Affiliations:** 1 Department of Biology, University of North Carolina at Chapel Hill, Chapel Hill, NC 27599; 2 Department of Cell Biology and Physiology, University of North Carolina at Chapel Hill, Chapel Hill, NC 27599; 3 Integrative Program for Biological and Genome Sciences, University of North Carolina at Chapel Hill, Chapel Hill, NC 27599

**Keywords:** dopamine, *Drosophila*, mushroom body, synaptic plasticity

## Abstract

The *Drosophila* mushroom body (MB) is an important model system for studying the synaptic mechanisms of associative learning. In this system, coincidence of odor-evoked calcium influx and dopaminergic input in the presynaptic terminals of Kenyon cells (KCs), the principal neurons of the MB, triggers long-term depression (LTD), which plays a critical role in olfactory learning. However, it is controversial whether such synaptic plasticity is accompanied by a corresponding decrease in odor-evoked calcium activity in the KC presynaptic terminals. Here, we address this question by inducing LTD by pairing odor presentation with optogenetic activation of dopaminergic neurons (DANs). This allows us to rigorously compare the changes at the presynaptic and postsynaptic sites in the same conditions. By imaging presynaptic acetylcholine release in the condition where LTD is reliably observed in the postsynaptic calcium signals, we show that neurotransmitter release from KCs is depressed selectively in the MB compartments innervated by activated DANs, demonstrating the presynaptic nature of LTD. However, total odor-evoked calcium activity of the KC axon bundles does not show concurrent depression. We further conduct calcium imaging in individual presynaptic boutons and uncover the highly heterogeneous nature of calcium plasticity. Namely, only a subset of boutons, which are strongly activated by associated odors, undergo calcium activity depression, while weakly responding boutons show potentiation. Thus, our results suggest an unexpected nonlinear relationship between presynaptic calcium influx and the results of plasticity, challenging the simple view of cooperative actions of presynaptic calcium and dopaminergic input.

## Significance Statement

Dopamine-induced long-term synaptic depression in the mushroom body (MB) is a key mechanism of olfactory associative learning in *Drosophila*. Although multiple lines of evidence indicate the presynaptic origin of this plasticity, it has been controversial whether and how the plasticity affects odor-evoked presynaptic calcium influx. Here, we demonstrate that the plasticity of presynaptic calcium signals is highly heterogeneous across individual presynaptic boutons, even among those on the same, likely spiking axons. The mode of plasticity (i.e., potentiation or depression) depends on the original response size. These results challenge the simple view that coincidence of presynaptic calcium and dopaminergic input triggers depression and suggest the plasticity outcome is finely controlled by the activity of individual synapses and local neuromodulatory signals.

## Introduction

Persistent changes in synaptic transmission are one of the fundamental mechanisms of learning. The mushroom body (MB), the learning and memory center of the *Drosophila* brain, serves as an important model for studying learning. Driven by genetic and circuit mapping of olfactory associative memory, studies in the MB have successfully bridged molecules, circuits, and behavior (Tomchik and Davis, 2013). However, the physiological characterization of synaptic plasticity itself lags behind other models, like the hippocampus (Mellor, 2018) and amygdala (Pape and Pare, 2010), where a legacy of *in vitro* experiments detailed the physiological events in synaptic plasticity. The field awaits dissection of the fundamental physiological elements underlying associative learning-related synaptic plasticity in the *Drosophila* MB.

**Figure 1.**
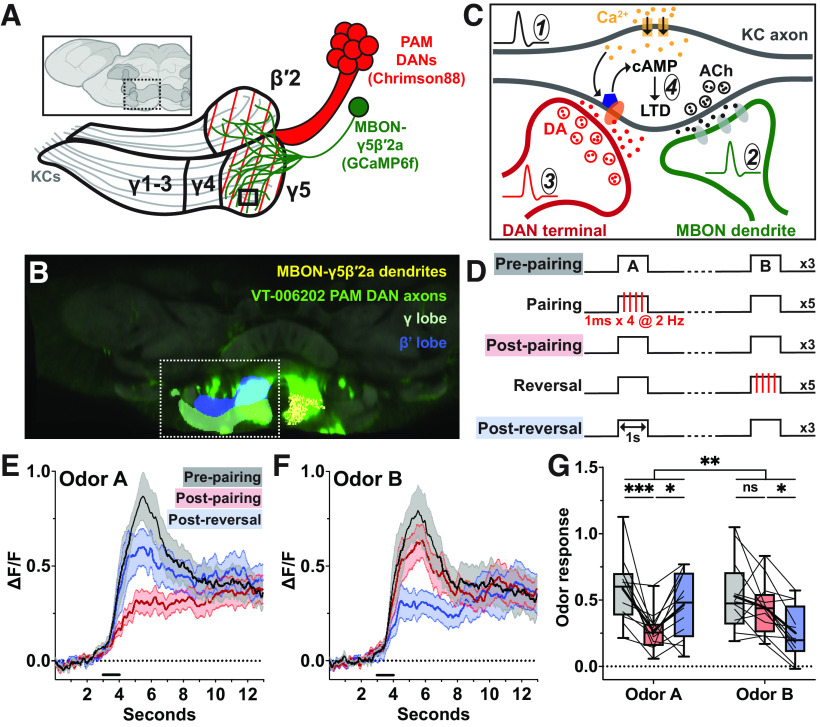
Ca^2+^ activity is depressed at the MBON-γ5β′2a dendrites following odor-DAN pairing. ***A***, Anterior view of *Drosophila* brain (via BioRender). Mushroom body colored dark gray. Hashed box marks location of illustration of Chrimson88-expressing subset of PAM DAN axons and GCaMP6f-expressing MBON-γ5β′2a dendrites. ***B***, Anterior view of the *Drosophila* brain (via Virtual Fly Brain) corresponding to ***A***. Left hemisphere, Blue, boundaries of β′ lobe; white, boundaries of γ lobe (Bogovic et al., 2020). Right hemisphere, Yellow, EM tracing skeleton of MBON-γ5β′2a dendrites (Xu et al., 2020). Both hemispheres: green: expression pattern of VT-006202 PAM DAN axons (Tirian and Dickson, 2017). ***C***, Conventional circuit model for associative olfactory learning; location corresponds to the solid black box in the γ compartment illustrated in ***A***. (1) A KC axon is activated by olfactory input, allowing Ca^2+^ (yellow dots) influx through voltage-gated Ca^2+^ channels (yellow rectangles). (2) Ca^2+^ influx promotes release of acetylcholine (black dots) via vesicular exocytosis, exciting the MBON dendrites. (3) Coincident DAN activation leads to activation of dopamine receptors (orange ellipsis) that activate rutabaga, a Ca^2+^-dependent adenylyl cyclase (blue pentagon). (4) Rutabaga synthesizes cAMP, initiating downstream signal transduction thought to promote synaptic plasticity. ***D***, Odor-DAN pairing protocol: pre-pairing, post-pairing, and post-reversal odor presentations are 1 s in duration separated by a 30-s interstimulus interval, repeated three times. Pairing and reversal each combine photostimulation (via 4 1-ms pulses delivered at 2 Hz) with Odor A and Odor B, respectively. ***E***, Odor A mean response profile (±SEM, shaded area; *N* = 12) at pre-pairing (black), post-pairing (red), and post-reversal (blue). Bold horizontal line indicates odor presentation. ***F***, Same as ***C*** but for Odor B. ***G***, Minimum-to-maximum box plots of mean ΔF/F of 5-s response window beginning at odor onset. Fine lines indicate data from individual flies; bold lines indicate mean; horizontal bar within box indicates median. Repeated-measures two-way ANOVA to compare effect of pairing across odors (interaction *p *=* *0.0044) and within odors at each stage of the experiment (Dunnett’s multiple comparison test, from left to right: *p *=* *0.0004, 0.0308, 0.4577, 0.0351). Asterisks indicate statistical significance: *p < 0.05, **p < 0.01, ***p < 0.001. “ns” indicates not significant, *p* > 0.05.

One of the strengths of the MB is its orderly circuit structure (Aso et al., 2014b). The lobes of the MB are made up of bundles of axons from odor identity-encoding Kenyon cells (KCs; Wang et al., 2004; Turner et al., 2008; Honegger et al., 2011). These lobes are precisely tiled by the dendrites of mushroom body output neurons (MBONs), which receive input from the KCs and communicate this odor-evoked signal to other brain regions to bias the fly’s odor-directed behavior (Aso et al., 2014a; Owald et al., 2015; Perisse et al., 2016). The lobes are similarly innervated by the axons of dopaminergic neurons (DANs), distinct subsets of which convey rewarding or punishing stimulus information (Schwaerzel et al., 2003; Schroll et al., 2006; Aso et al., 2010; Burke et al., 2012; C. Liu et al., 2012) to both KC axons and MBON dendrites (Takemura et al., 2017). Thus, the symmetrical innervation of the MB lobes by MBON dendrites and DAN axons define anatomic compartments (Fig. 1*A*) that act as functional units. Behavioral genetics studies have identified critical molecular components of this circuit required for learning, including a dopamine receptor (Kim et al., 2007; Qin et al., 2012) and a calcium (Ca^2+^)-dependent adenylate cyclase (Livingstone et al., 1984; McGuire et al., 2003; Mao et al., 2004). Since the essential expression site of these learning-related genes is KCs, those results led to the current working model that the coincidence of odor-evoked Ca^2+^ activity and dopaminergic input at the presynaptic KC axon terminal engages the cAMP signaling pathway, leading to plasticity at the KC-to-MBON synapses (Busto et al., 2010; Fig. 1*C*). In support of this model, simultaneous activation of KCs and DANs induces robust long-term depression (LTD) at those synapses (Berry et al., 2018; Cohn et al., 2015; Hige et al., 2015; Handler et al., 2019). This direction of plasticity can also explain the bias of behavior after learning because the stimulus valence signaled by a given DAN is generally opposite to the valence signaled by the MBONs of the same compartment (Aso et al., 2014a). However, direct evidence for the basic physiology underlying this plasticity, especially on the presynaptic side, is minimal and often debated.

Despite multiple lines of evidence supporting dopamine-induced LTD (Okada et al., 2007; Séjourné et al., 2011; Cohn et al., 2015; Hige et al., 2015; Owald et al., 2015; Berry et al., 2018; Handler et al., 2019), memory traces observed at KC axons have not necessarily been in line with depression. That is, there is varying *in vivo* imaging evidence for odor-evoked Ca^2+^ activity in the KC presynaptic terminals being depressed (Zhang et al., 2019), potentiated (Yu et al., 2006; Wang et al., 2008; Akalal et al., 2010; Boto et al., 2014, 2019; Louis et al., 2018), and unchanged (Louis et al., 2018) after learning, with the majority of reports supporting potentiation. The first pioneering studies to image odor-evoked Ca^2+^ activity in an immobilized fly before and after odor-shock pairing revealed post-pairing potentiation of odor responses (Yu et al., 2006; Wang et al., 2008). Similarly, pairing of odor with artificial activation of punishment-encoding DANs also led to potentiated odor responses, and pairing with artificial activation of reward-encoding DANs likewise led to potentiation (Boto et al., 2014, 2019). Postlearning potentiation of KCs is also supported by the observations that the anterior paired lateral (APL) neurons, a pair of GABAergic neurons innervating the entire MB neuropil, show odor-specific suppression after odor-shock pairing and that reduction of GABA synthesis in the APL neurons enhances learning (X. Liu and Davis, 2009; but also see Lin et al., 2014). However, other studies using training via pairing of odor with shock or sugar describe depressed (Zhang et al., 2019) and unchanged odor responses after aversive training alongside potentiated responses after appetitive training (Louis et al., 2018). While these disagreements could be because of subtle differences in experimental design or genetic tools used for artificial activation of DANs, the apparent lack of consistency and likely involvement of neuromodulators other than dopamine in natural learning makes it difficult to conclude whether dopamine-induced plasticity itself is accompanied by corresponding changes in presynaptic Ca^2+^ signaling.

In this study, we examined the effects of DAN-induced plasticity on the presynaptic side of the KC-to-MBON synapse by using optogenetic control of DANs with compartment specificity to isolate the effect of DAN activation while enabling direct comparison with postsynaptically observed plasticity. After establishing the condition in which paired odor presentation and DAN activation (“odor-DAN pairing”) induces reliable and odor-specific LTD, we applied this condition to complete a systematic inspection of activity along several key sites of KC-to-MBON transmission. This strategy revealed compartment-specific depression of neurotransmitter release by KCs, but Ca^2+^ dynamics of the KC axon bundles remained unaffected. However, further inspection of Ca^2+^ dynamics with the resolution of individual presynaptic boutons exposed an underlying mixture of bidirectional changes scattered across boutons. Not only does this result challenge our understanding of the interplay of Ca^2+^ and dopamine, but it also opens the way for future mechanistic explorations of this important model of associative learning.

## Materials and Methods

### Flies

All flies were raised at room temperature (20–21°C) on standard cornmeal-agar-based food (glucose fly food, Archon Scientific). At 1–2 d posteclosion, flies for experiments were selected based on sex and genotype after sorting under brief CO_2_ anesthesia. All experiments used female flies that fed on standard food supplemented with all-trans-retinal (R2500, Sigma-Aldrich) at a final concentration of 0.5 mm. Flies fed on retinal-supplemented food in the dark for at least 48 h before experiments. At the time of experiment, all flies were at an age of 3–6 d posteclosion. All fly genotypes are summarized in Table 1.

**Table 1:** Experimental fly genotypes

Figure	Genotype
Figure 1*C–E*	20XUAS-IVS-Syn21-OpGCaMP6f-p10(attP8); VT006202-LexA(attP40); 13×LexAop2-IVS-Syn21-Chrimson88:tdT-3.1-p10(VK00005)/SS01308-split-GAL4
Figure 2*B–D*	VT006202-LexA(attP40); 13×LexAop2-IVS-Syn21-Chrimson88:tdT-3.1-p10(VK00005)/R13F02-GAL4(attP2), UAS-ACh3.0(VK00005)
Figure 3*B–D*	20XUAS-IVS-Syn21-OpGCaMP6f-p10(attP8); VT006202-LexA(attP40); 13×LexAop2-IVS-Syn21-Chrimson88:tdT-3.1-p10(VK00005)/R13F02-GAL4(attP2)
Figure 4*B*	20XUAS-IVS-Syn21-OpGCaMP6s-p10(attP8); VT45661-LexA(JK22C); 13×LexAop2-IVS-Syn21-Chrimson88:tdT-3.1-p10(VK00005)/MB112C-split-GAL4
Figure 4*C,D*	VT45661-LexA(JK22C); 13×LexAop2-IVS-Syn21-Chrimson88:tdT-3.1-p10(VK00005)/R13F02-GAL4(attP2), UAS-ACh3.0(VK00005)
Figure 4*E,F*	20XUAS-IVS-Syn21-OpGCaMP6f-p10(attP8); VT45661-LexA(JK22C); 13×LexAop2-IVS-Syn21-Chrimson88:tdT-3.1-p10(VK00005)/R13F02-GAL4(attP2)
Figures 5*C–K*, 6*A–F*, 7*A–F*; Extended Data Figures 5-1*A–F*, 6-1*A–F* (“Paired”)	20XUAS-IVS-phiC31(attP18); VT006202-LexA(attP40)/20×UAS-SPARC-S-jGCaMP7f(CR-P40); 13×LexAop2-IVS-Syn21-Chrimson88:tdT-3.1-p10(VK00005)/R13F02-GAL4(attP2)
Extended Data Figure 6-1*A–F* (“G.C.”)	20XUAS-IVS-phiC31(attP18); 20×UAS-SPARC-S-jGCaMP7f(CR-P40); 13×LexAop2-IVS-Syn21-Chrimson88:tdT-3.1-p10(VK00005)/R13F02-GAL4(attP2)

The genotype of each experimental fly is reported according to the corresponding data, listed by figure panel.

### *In vivo* two-photon imaging

Imaging experiments were conducted using a 930 nm, femtosecond fiber laser (YLMO-930, MenloSystems) and a moveable objective microscope (Sutter Instruments) fitted with a 20× and 1.00 NA water immersion objective (XLUMPLFLN, Olympus) and PMTs (H11706P-40, Hamamatsu) and controlled by ScanImage 5.6 software (Pologruto et al., 2003; Vidrio Technologies). Flies were prepared for *in vivo* imaging by a dissection procedure that removes a dorsal section of the fly’s head capsule cuticle to gain optical access to the brain. Briefly described, the fly was anesthetized on ice then fixed in place with UV-curing glue (Loctite AA 3972, Henkel Adhesives) on a custom-built dissection plate. The dissection is then conducted under saline (275 mOsm) containing NaCl (103 mm), KCl (3 mm), TES (5 mm), CaCl_2_ (1.5 mm), MgCl_2_ (4 mm), NaH_2_PO_4_ (1 mm), NaHCO_3_ (26 mm), glucose (10 mm), and trehalose (10 mm), held at a pH of 7.3 via carbogen bubbling. The overlying cuticle and any fat bodies or air sacs that disrupted the imaging path were removed. After muscles #1 and #13 (Schwarz et al., 2017) were disabled to prevent proboscis extension-induced brain motion, the fly was transferred to the imaging rig with continued perfusion of carbogen-bubbled saline. The fly was allowed to habituate for 10 min before the experiment began.

#### Ca^2+^ and ACh imaging of MBON dendrites and bulk KC axons

**Figure 2.**
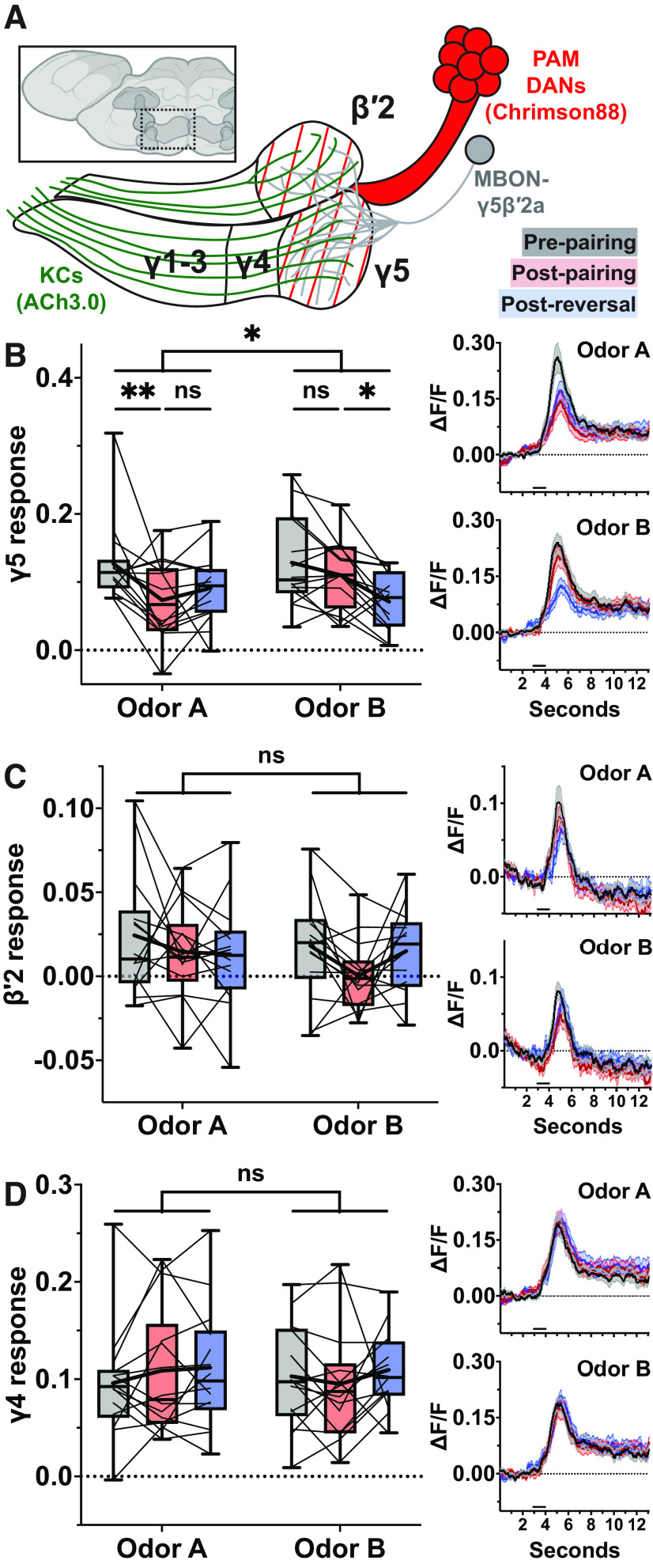
Odor-evoked acetylcholine release is depressed at the mushroom body lobes with compartment specificity following odor-DAN pairing. ***A***, Anterior view of *Drosophila* brain. Mushroom body colored dark gray. Hashed box marks location of illustration of Chrimson88-expressing subset of PAM DAN axons and ACh3.0-expressing KC axons. ***B***, Minimum-to-maximum box plots of mean ΔF/F of response window of axons within γ5 compartment (*N* = 14). Fine lines indicate data from individual flies; bold lines indicate mean; horizontal bar within box indicates median. Repeated-measures two-way ANOVA to compare effect of pairing across odors (interaction *p *=* *0.0390) and within odors at each stage of the experiment (Dunnett’s multiple comparison test, from left to right: *p *=* *0.0020, 0.4176, 0.3889, 0.0265). Right, Upper, Odor A mean response profile (±SEM, shaded area) from γ5 compartment at pre-pairing (black), post-pairing (red), and post-reversal (blue). Right, Lower, Same as upper but for Odor B. Typical field of view and selection of ROIs are shown in Extended Data Figure 2-1. ***C***, Same as ***B*** but for β′2 compartment (*N* = 14). Effect of pairing across odors (interaction *p *=* *0.4433). ***D***, Same as ***B*** but for γ4 compartment (*N* = 14). Effect of pairing across odors (interaction *p *=* *0.5274). Asterisks indicate statistical significance: *p < 0.05, **p < 0.01. “ns” indicates not significant, *p* > 0.05.

**Figure 3.**
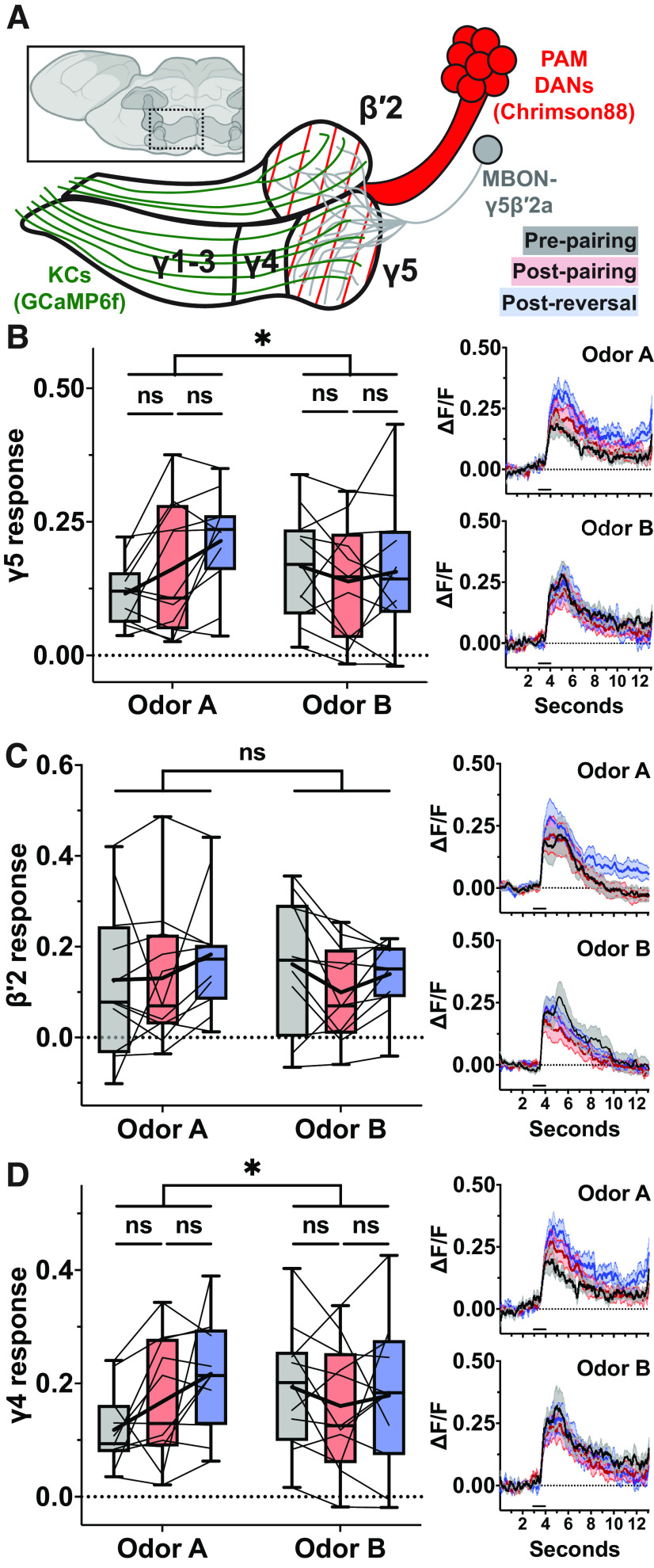
Bulk Ca^2+^ activity of KC axons does not change following odor-DAN pairing. ***A***, Anterior view of *Drosophila* brain. Mushroom body colored dark gray. Hashed box marks location of illustration of Chrimson88-expressing subset of PAM DAN axons and GCaMP6f-expressing KC axons. ***B***, Minimum-to-maximum box plots of mean ΔF/F of response window of axons within γ5 compartment (*N* = 11). Fine lines indicate data from individual flies; bold lines indicate mean; horizontal bar within box indicates median. Repeated-measures two-way ANOVA to compare effect of pairing across odors (interaction *p *=* *0.0227) and within odors at each stage of the experiment (Dunnett’s multiple comparison test, from left to right: *p *=* *0.1668, 0.1129, 0.4829, 0.7307). Right, Upper, Odor A mean response profile (±SEM, shaded area) from γ5 compartment at pre-pairing (black), post-pairing (red), and post-reversal (blue). Right, Lower, Same as upper but for Odor B. ***C***, Same as ***B*** but for β′2 compartment (*N* = 11). Effect of pairing across odors (interaction *p *=* *0.1477). ***D***, Same as ***B*** but for γ4 compartment (*N* = 11). Effect of pairing across odors (interaction *p *=* *0.0409) and within odors at each stage of the experiment (Dunnett’s multiple comparison test, from left to right: *p *=* *0.2121, 0.2212, 0.4735, 0.7854). Asterisks indicate statistical significance: *p < 0.05. “ns” indicates not significant, *p* > 0.05.

**Figure 4.**
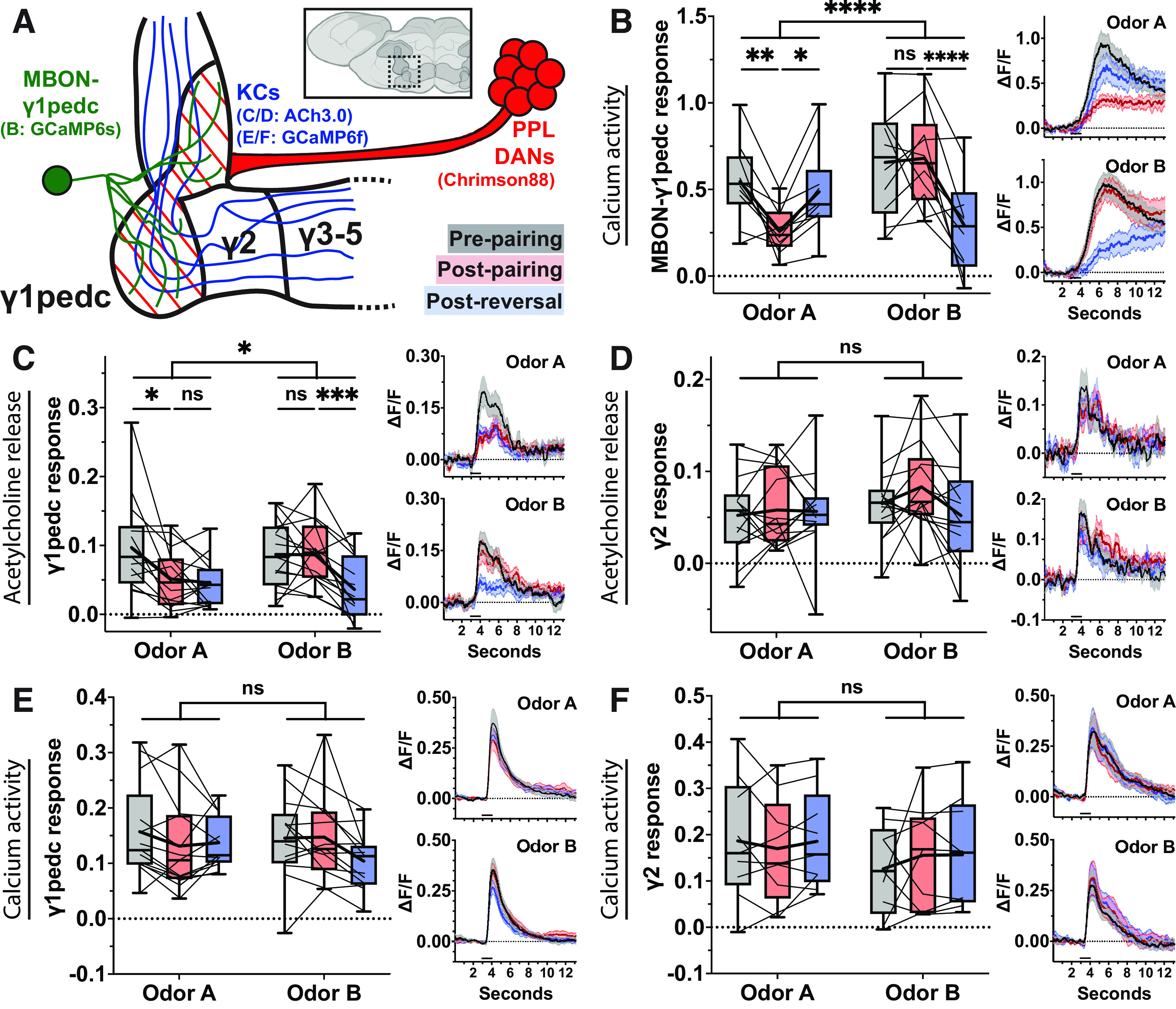
Effects of odor-DAN pairing at MBON-γ1pedc and relevant MB compartments. ***A***, Anterior view of *Drosophila* brain. Mushroom body colored dark gray. Hashed box marks location of illustration of Chrimson88-expressing subset of PPL1 DAN axons and sensor-expressing neurites: corresponding with ***B***, GCaMP6s-expressing dendrites of MBON-γ1pedc; corresponding with ***C***, ***D***, ACh3.0-expressing KC axons; corresponding with ***E***, ***F***, GCaMP6f-expressing KC axons. ***B***, Minimum-to-maximum box plots of mean ΔF/F of response window of MBON-γ1pedc dendrites (*N* = 10). Fine lines indicate data from individual flies; bold lines indicate mean; horizontal bar within box indicates median. Repeated-measures two-way ANOVA to compare effect of pairing across odors (interaction *p *<* *0.0001) and within odors at each stage of the experiment (Dunnett’s multiple comparison test, from left to right: *p *=* *0.0022, 0.0149, 0.9348, <0.0001). Right, Upper, Odor A mean response profile (±SEM, shaded area) from MBON-γ1pedc dendrites at pre-pairing (black), post-pairing (red), and post-reversal (blue). Right, Lower, Same as upper but for Odor B. ***C***, Same as ***B*** but for ACh release in γ1pedc compartments (*N* = 15). Effect of pairing across odors (interaction *p *=* *0.0290) and within odors at each stage of the experiment (Dunnett’s multiple comparison test, from left to right: *p *=* *0.0167, 0.7553, 0.9986, 0.0006). ***D***, Same as ***C*** but for γ2 compartment (*N* = 14). Effect of pairing across odors (interaction *p *=* *0.2616). ***E***, Same as ***B*** but for Ca^2+^ activity in γ1pedc compartments (*N* = 13). Effect of pairing across odors (interaction *p *=* *0.1207). ***F***, Same as ***E*** but for γ2 compartment (*N* = 9). Effect of pairing across odors (interaction *p *=* *0.5037). Asterisks indicate statistical significance: *p < 0.05, **p < 0.01, ***p < 0.001, ****p < 0.0001. “ns” indicates not significant, *p* > 0.05.

**Figure 5.**
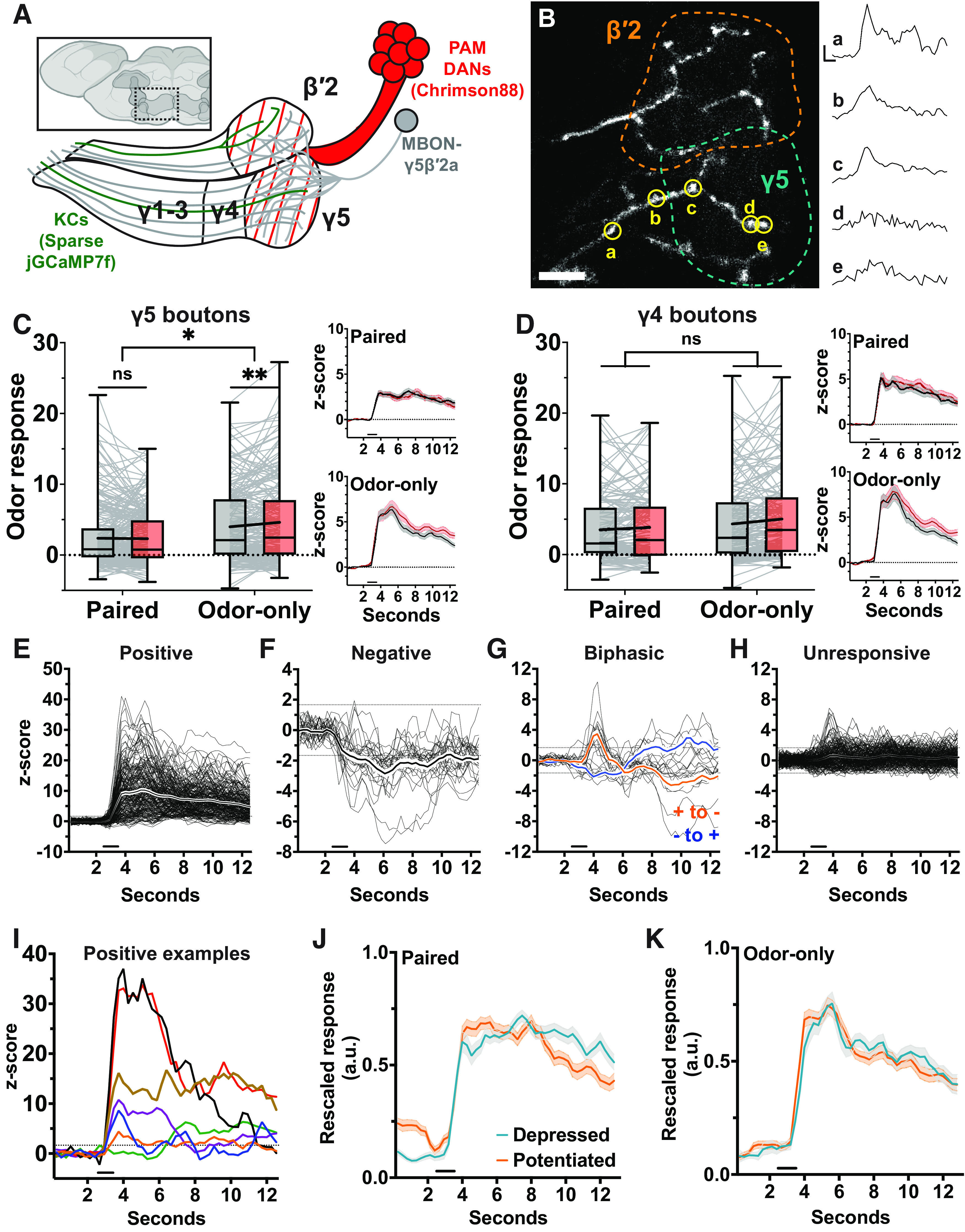
Diverse effects of odor-DAN pairing on Ca^2+^ activity of individual presynaptic boutons of KCs. ***A***, Anterior view of *Drosophila* brain. Mushroom body colored dark gray. Hashed box marks location of illustration of Chrimson88-expressing subset of PAM DANs and KCs sparsely expressing jGCaMP7f. ***B***, Left, Maximum z-projection of a typical field of view from a bouton imaging experiment; γ5 outlined in teal, β′2 in orange; scale bar, 5 μm; five example boutons circled in yellow (a-e). Right, Response profiles of each example bouton; scale bar, 5 s and *z* score of 5. ***C***, Minimum-to-maximum boxplots of mean *z* score of the 10-s window following odor presentation for all boutons within the γ5 compartment (paired *n* = 263 boutons, *N* = 10 flies; odor-only *n* = 222, *N* = 11). Fine lines indicate data from individual flies; bold lines indicate mean; horizontal bar within box indicates median. Repeated-measures two-way ANOVA to compare effect of pairing across odors (interaction *p *=* *0.0125) and within odors at each stage of the experiment (Šídák’s multiple comparisons test, from left to right: *p *=* *0.9100, 0.0048). Right, Upper, Paired odor mean response profile (±SEM, shaded area) at pre-pairing (black) and post-pairing (red). Right, Lower, Same as upper but for odor-only condition. ***D***, Same as ***C*** but for all boutons within the γ4 compartment (paired *n* = 150, *N* = 11; odor-only *n* = 178, *N* = 10). Repeated-measures two-way ANOVA to compare effect of pairing across odors (interaction *p *=* *0.3038). ***E–H***, All γ5 boutons are divisively filtered into groups based on conservative thresholds applied to their individual response profiles. Positive (***E***, *n* = 251; mean total *z* score > 1.6449, corresponding to *p *=* *0.05 threshold), negative (***F***, *n* = 30; ≥1 s of mean *z* score > 1.6449), biphasic (***G***, *n* = 25; ≥1 s of mean *z* score > 1.6449 and < 1.6449), and unresponsive (*n* = 179; all remaining boutons). ***I***, Example mean pre-pairing responses taken from seven boutons categorized as “Positive” to illustrate within-group diversity. ***J***, Rescaled pre-pairing responses to paired odor in boutons that will undergo changes of at least 20% after pairing, either via depression (*n* = 52; blue line) or potentiation (*n* = 51; orange line). ***K***, Same as ***I*** but for the odor-only condition (will be depressed, *n* = 37; will be potentiated, *n* = 66). Further analyses based on temporal characteristics of γ5 bouton responses are shown in Extended Data Figure 5-1. Asterisks indicate statistical significance: *p < 0.05, **p < 0.01. “ns” indicates not significant, *p* > 0.05.

**Figure 6.**
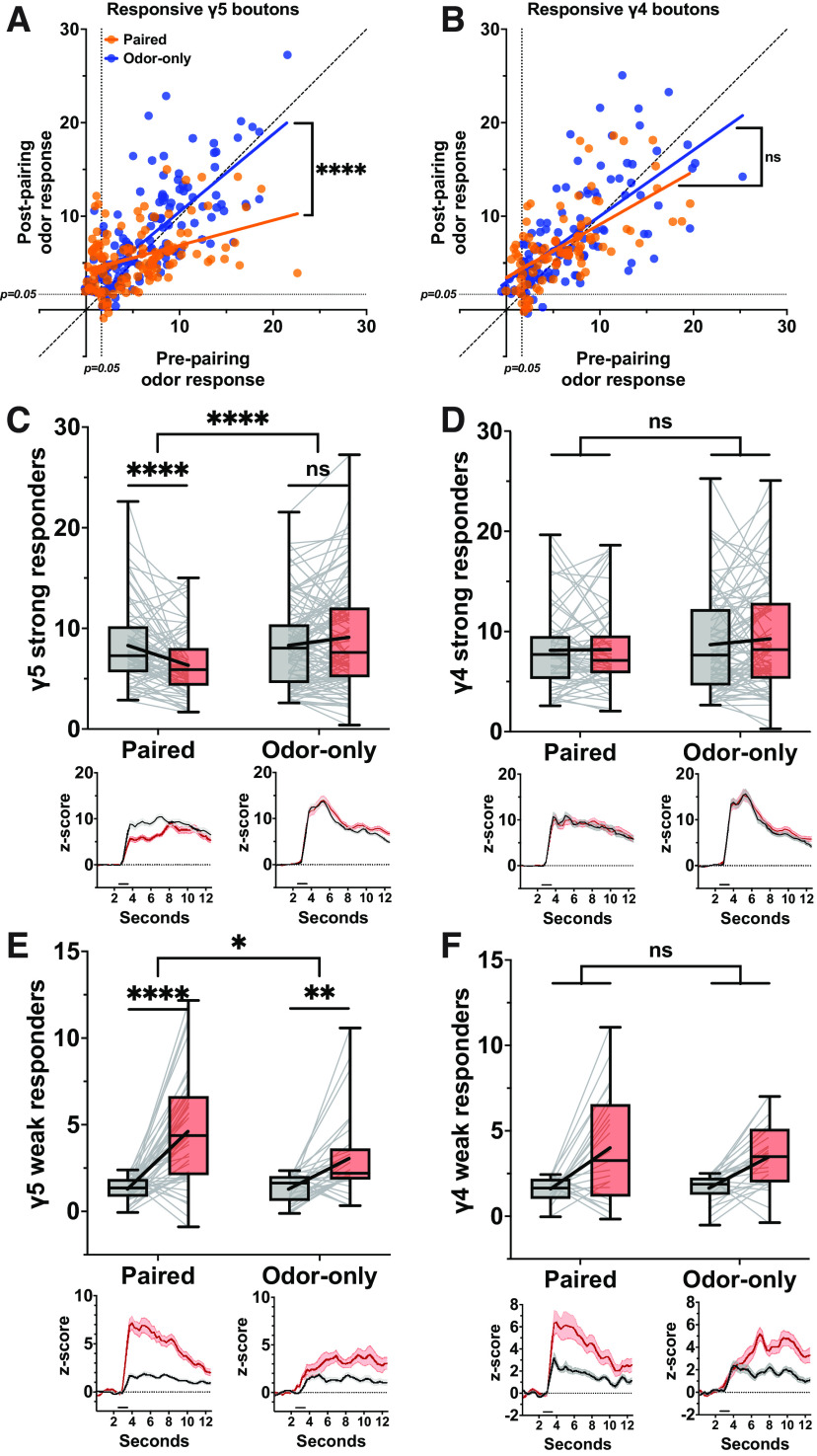
Plasticity of γ5 boutons is bidirectionally biased by the magnitude of naive odor responses. ***A***, Pre-pairing mean *z* score plotted against post-pairing mean *z* score for all γ5 boutons responsive to odor at either pre-pairing or post-pairing. Orange points represent paired odor responsive boutons (*n* = 116); blue points represent odor-only condition responsive boutons (*n* = 135); solid lines represent linear regression lines compared by ANCOVA (*p *<* *0.0001); hashed black line marks line of identity. ***B***, Same as ***A*** but for odor responsive boutons in γ4 compartment (paired *n* = 84, odor-only *n* = 111); linear regression lines compared via ANCOVA (*p *=* *0.2176). ***C***, Minimum-to-maximum box plots of mean *z* score of “strong responder” boutons within γ5 compartment (paired *n* = 71, odor-only *n* = 105). Fine lines indicate data from individual flies; bold lines indicate mean; horizontal bar within box indicates median. Repeated-measures two-way ANOVA to compare effect of pairing across odors (interaction *p *<* *0.0001) and within odors at each stage of the experiment (Šídák’s multiple comparisons test, from left to right: *p *<* *0.0001, *p* = 0.0775). Lower, Left, Paired odor mean response profile (±SEM, shaded area) at pre-pairing (black), post-pairing (red), and post-reversal (blue). Lower, Right, Same as left but for odor-only condition. ***D***, Same as ***C*** but for “strong responder” boutons in γ4 compartment (paired *n* = 61, odor-only *n* = 85). Repeated-measures two-way ANOVA to compare effect of pairing across odors (interaction *p *=* *0.4769). ***E***, Same as ***C*** but for “weak responder” boutons in γ5 compartment (paired *n* = 45, odor-only *n* = 30). Repeated-measures two-way ANOVA to compare effect of pairing across odors (interaction *p *=* *0.0305) and within odors at each stage of the experiment (multiple comparisons from left to right: *p *<* *0.0001, 0.0037). ***F***, Same as ***B*** but for “weak responder” boutons in γ4 compartment (paired *n* = 23, odor-only *n* = 26). Repeated-measures two-way ANOVA to compare effect of pairing across odors (interaction *p *=* *0.4380). We also performed pairing experiments in genetic control flies, and the data are shown in Extended Data Figure 6-1. Asterisks indicate statistical significance: *p < 0.05, **p < 0.01, ***p < 0.0001. “ns” indicates not significant, *p* > 0.05.

**Figure 7.**
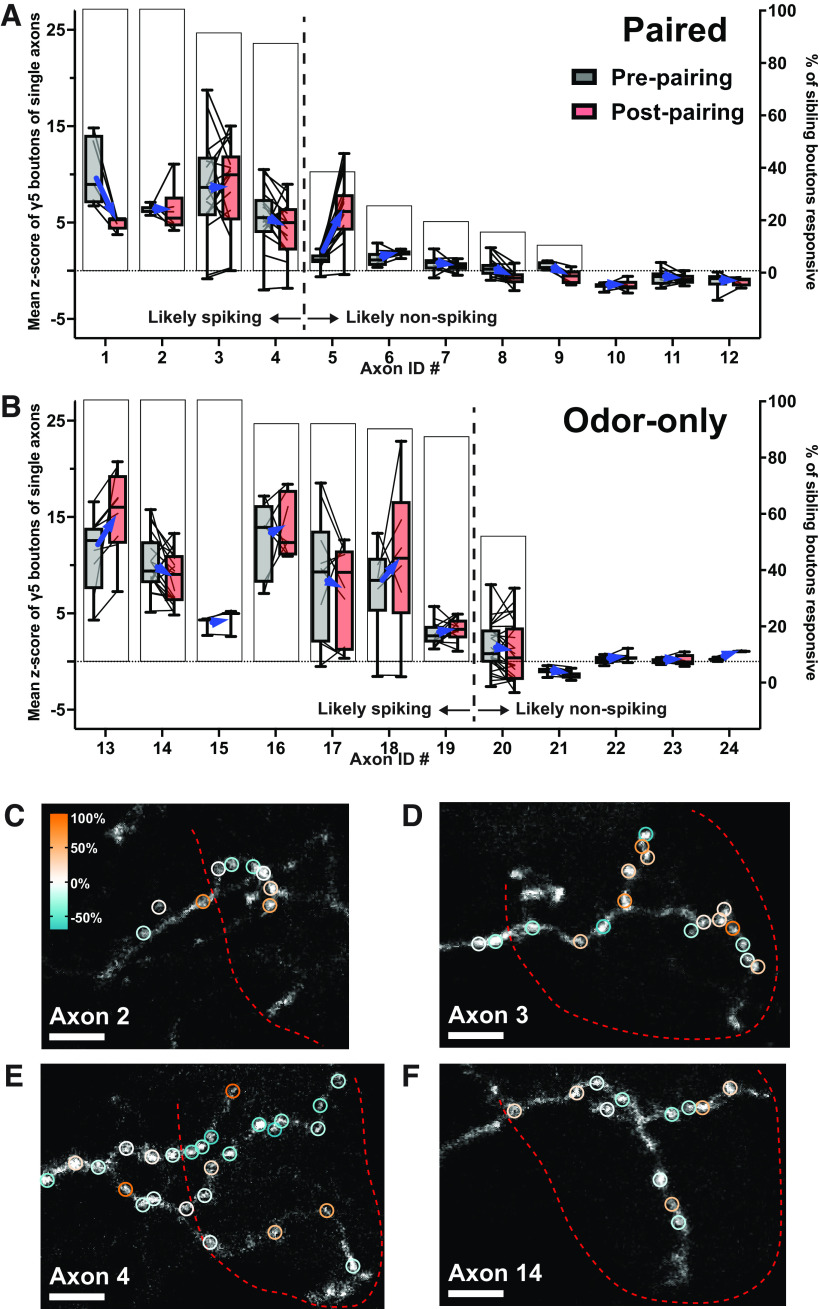
Heterogeneous plasticity exhibited by boutons on likely-spiking axons. ***A***, Pre-pairing and post-pairing mean total *z* score of paired γ5 boutons (left *y*-axis) grouped according to their parent axons (1–12 on *x*-axis). White bar graph (right *y*-axis) denotes the percentage of boutons on the parent axon that responds to odor. The hashed vertical line separates “likely spiking axons” from “likely nonspiking axons” based on an 80% threshold. ***B***, Same as ***A*** but for odor-only γ5 boutons. ***C***, Maximum z-projection of axon #2, which is likely spiking in response to odor that was paired with odor-DAN activation. Bouton ROIs are marked by open circles whose color corresponds to the percent change of their naive odor response after pairing. Red hashed line marks boundary around the γ5 compartment; axon to the left of the bounded area belongs to the γ4 compartment; left to right is proximal to distal; scale bar is 3 μm. ***D***, Same as ***C*** but for axon #3. ***E***, Same as ***C*** but for axon #4. ***F***, Same as ***C*** but for axon #14, which is likely spiking in response to odor that was presented in odor-only condition.

For the experiments examining the γ5 and β′2 compartments (Figs. 1–3 and 5–7), the field of view was centered on the distal tip of the horizontal lobe, guided by fluorescence of the Ca^2+^ or ACh sensor expressed by the target cell type and by tdTomato-tagged Chrimson expressed by DANs (via VT-006202-LexA) innervating the γ5 and β′2 compartments. These compartments as well as the γ4 compartment (which served as an internal control) were visible in all experiments. All Ca^2+^ imaging experiments used GCaMP6f as the Ca^2+^ sensor. Imaging frames were collected at 512 × 512 pixels and ∼30 Hz with a laser power of ∼15 mW for bulk KC Ca^2+^ imaging and ∼35 mW for MBON dendrite Ca^2+^ imaging and bulk KC ACh imaging. For the experiments examining the γ1 compartment (Fig. 4), the field of view was centered on the MB heel, guided by fluorescence of the Ca^2+^ or ACh sensor expressed by the target cell type and by tdTomato-tagged Chrimson expressed by DANs (VT-45 661-LexA) innervating the γ1 compartment and peduncle subregions. These compartments were visible in all experiments, and the γ2 compartment (which served as an internal control) was also visible in most. KC Ca^2+^ imaging experiments used GCaMP6f as the Ca^2+^ sensor while MBON-γ1pedc dendrite imaging used GCaMP6s because the baseline signal of GCaMP6f in MBON-γ1pedc (via MB112C split-GAL4 driver) was not strong enough for consistent identification. Imaging frames were collected at 512 × 512 pixels and ∼15 Hz with a laser power of ∼15 mW for bulk KC Ca^2+^ imaging and ∼35 mW for MBON dendrite Ca^2+^ imaging and bulk KC ACh imaging.

#### Ca^2+^ imaging of sparsely labeled KC axons

The field of view was centered on the distal tip of the horizontal lobe, as in preceding experiments targeting this brain region. Volumetric imaging was used to capture the full anatomy of KC axons sparsely expressing jGCaMP7f. We used jGCaMP7f instead of GCaMP6f for these experiments because jGCaMP7f gave a higher basal fluorescence intensity in our preliminary experiments, which was advantageous for imaging small structures. Volumes were collected in seven slices with 3-μm steps per slice and at a volume rate of ∼3.75 Hz. Imaging frames were collected at 512 × 512 pixels and ∼25-mW laser power. An 8-odor panel was used to find an odor that activated the jGCaMP7f-expressing axon(s) within the imaging volume. When a response-evoking odor was identified, the panel was stopped, the response-evoking odor was selected to serve as Odor A, and one of the nonresponse-evoking odors was selected to serve as Odor B.

### Stimulus delivery

A custom-designed 8-odor delivery system, controlled by in-house MATLAB routines, was used for odor presentation. The odor panel contained vials loaded with 5 ml of: 4-methylcyclohexanol (MCH; 166902, Acros Organics), 3-octanol (OCT; O0121, TCI Chemicals), 2-heptanone (A10200, Alfa Aesar), isoamyl acetate (150662, Acros Organics), ethyl lactate (W244015, Sigma-Aldrich), ethanol (BP2818, Acros Organics), yeast (1 g mixed in 1 ml of water; Baker’s Dry Yeast, Saf-Instant), and apple cider vinegar (Distilled, Heinz). Saturated vapor of odor was air-diluted to 1% and presented to the fly head-on, and air flow was held at a constant 4.2 m/s. All pre-pairing, post-pairing/reversal, pairing/reversal odor presentations were 1 s in duration. Tight temporal control of odor presentation was confirmed by a photoionization detector (200B mini PID, Aurora Scientific). Pre-pairing and post-pairing/reversal odor exposures were presented with a 30-s interstimulus interval; pairing/reversal odor exposures were presented with a 60-s interstimulus interval. A 625-nm LED (M625L3, Thorlabs) and LED driver (LEDD1B, Thorlabs), externally controlled by the same custom MATLAB routine that directed the odor delivery system, was used to deliver photostimulation through the objective lens at 30 mW/mm^2^ during pairing/reversal. Four pulses of 1-ms duration were delivered at 2 Hz, starting at ∼300 ms after odor onset. In the half of experiments assigned to the “odor-only” condition, the LED was unpowered during odor-DAN pairing. We used odor-DAN pairing protocols similar to those used in behavioral experiments that activated the same or similar DAN subpopulations (Aso and Rubin, 2016; Yamada et al., 2023). Although we did not test whether our exact training protocols can induce behavioral changes, they successfully induced changes in olfactory responses in the MBONs that are consistent with previously reported learning-related changes.

### Data analysis

#### Ca^2+^ and ACh imaging of MBON dendrites and bulk KC axons

At the conclusion of each imaging experiment, a high-resolution z-stack was taken of the volume surrounding the experimental imaging plane. This was used to guide region of interest (ROI) selection during image analysis. Before ROI selection, translational rigid motion was corrected using the ImageJ plugin, *moco* (Dubbs et al., 2016). For MBON dendrite imaging, single ROIs were drawn at the main trunk of the dendrite, downstream of the fine arborizations in the MB compartments, and for bulk KC imaging, ROIs were drawn around individual compartments with the guidance of tdTomato-tagged Chrimson expressed by DAN axons and the gross anatomic features of the MB lobes. ROIs were drawn on the average of the motion corrected images from the entire experiment using a modified version of an open access imaging processing toolbox (Romano et al., 2017). ROI boundaries were drawn conservatively, inside the actual compartment boundaries identified using GFP-based sensor and tdTomato-tagged opsin signals (Cohn et al., 2015). Subsequent analysis of fluorescence intensity was completed using custom-written MATLAB routines. The ΔF/F was calculated using the 2 s before the odor stimulus trigger as a baseline. To quantify the odor response, the mean ΔF/F was calculated for the response window. The response window was defined as the 5-s period following odor onset, except for in the case of Ca^2+^ imaging of MBON-γ1pedc dendrites. To account for the slower Ca^2+^ sensor (GCaMP6s), the response window in this experiment was extended from 5 to 10 s.

#### Ca^2+^ imaging of sparsely labeled KC axons

A high-resolution z-stack was taken at the conclusion of each experiment, as mentioned above. It was used to guide ROI selection and to assign boutons to their parent axons. Each volume slice was preprocessed and analyzed independently. Because of the weak sensor signal at baseline, necessary rigid motion correction was detected using preprocessed stacks (three-pixel maximum 3D filter followed by three-pixel mean 2D filter) then applied to raw images that were used for further analysis. Because of frequent nonrigid drift, this motion correction strategy was typically only sufficient to align images within each period (pre-pairing, post-pairing/reversal) of the experiment. Therefore, ROIs were drawn on the mean of the slice at each period of the experiment separately then unified later. Boutons were detected based on the easily identifiable axonal swellings, areas of greater signal strength and width relative to the surrounding axonal shaft, as previously validated (Bilz et al., 2020). Subsequent analysis of fluorescence intensity was completed using custom-written MATLAB routines. The previously mentioned weak sensor signal made the ΔF/F an unreliable measure, so each bouton’s response profile was described based on its own baseline-defined *z* score. The *z* score for each time point was calculated as the raw intensity value less the mean of the baseline period (2 s preceding odor stimulus trigger), divided by the standard deviation of the baseline period. To include the diversity of response (Fig. 5*I*), the response window in these experiments is defined as 10 s.

### Statistical tests and figure design

Statistical tests were completed with GraphPad Prism (9.5.1). Our statistics procedure was to compare the odor response (quantified via mean ΔF/F of the response window in Figs. 1–4 and as mean *z* score of response window in Figs. 5–7) of each odor at every stage of the experiment (pre-pairing, post-pairing, post-reversal) via repeated-measures two-way ANOVA then, in cases where the interaction *p* value indicates a significantly different effect of pairing between odors, further compare within odors and across stages of the experiment using Dunnett’s multiple comparison test (for pre-pairing, post-pairing, and post-reversal comparisons) or using Šídák’s multiple comparison test (for pre-pairing and post-pairing comparisons). Figure design was completed with a combination of MATLAB (2020b) and Adobe Illustrator (27.5). For presentation, all example images were processed with a 0.5-pixel median filter. All data generated in this work will be shared on reasonable request to the corresponding author.

## Results

### Odor-DAN pairing induces odor-specific suppression of responses in MBON-γ5β′2a

Our fundamental strategy for this study is to systematically test, under identical conditions, the expression of synaptic plasticity at a series of sites along the KC-to-MBON transmission pathway. To this end, we first tested the effect of odor-DAN pairing on the postsynaptic side. We primarily focused on MBON-γ5β′2a (also known as MBON-01 and MB-M6; Aso et al., 2014a) since relative proximity of its dendritic arborization to the anterior surface of the brain allows for detailed imaging of its partner presynaptic boutons (see below). MBON-γ5β′2a primarily receives input from KC axons of the γ5 compartment and secondarily from the anterior region of the β′2 compartment (Aso et al., 2014b). The DAN axons that innervate these compartments originate in the protocerebral anterior medial (PAM) DAN cluster, known to be involved in associative learning. Despite numerous reports about this MBON and the corresponding DANs’ involvement in a variety of functions, including appetitive learning (Aso et al., 2014a; Huetteroth et al., 2015; Owald et al., 2015; Yamagata et al., 2015), food-seeking behavior (Tsao et al., 2018), courtship learning (Keleman et al., 2012; Zhao et al., 2018), and sleep homeostasis (Sitaraman et al., 2015), plasticity induced by odor-DAN pairing has not been directly observed. We therefore set out to address this issue by using *in vivo* two-photon Ca^2+^ imaging of the MBON-γ5β′2a dendrites together with optogenetic activation of DANs. We exclusively expressed a Ca^2+^ sensor (GCaMP6f; Chen et al., 2013) in MBON-γ5β′2a using a split-GAL4 driver (SS01308) and drove expression of a red light-gated cation channel (Chrimson88; Strother et al., 2017) in associated DANs using VT-006202-LexA (Fig. 1*A*). This LexA driver targets the γ5 and β′2 compartments with additional weak labeling in the γ3 compartment (Fig. 1*B*; Zhao et al., 2018; Court et al., 2023).

We measured Ca^2+^ responses to two odors, Odors A and B, before and after pairing Odor A with photostimulation of DANs (Fig. 1*D*, “Pairing”). Subsequently, Odor B was paired with photostimulation (Fig. 1*D*, “Reversal”), and we measured the responses again. In half of the experiments, Odor A was 4-methylcyclohexanol (MCH), and Odor B was 3-octanol (OCT). The odors were swapped in the other half of the experiments to eliminate any odor-specific bias. After the first round of pairing, we observed a depressed response to Odor A compared with no detectable change to the Odor B response (Fig. 1*E–G*). After the second round of pairing, we found that the response to Odor A was potentiated, suggesting recovery, while the response to Odor B was depressed (Fig. 1*E–G*). These results demonstrate that odor-DAN pairing can induce robust odor-specific depression in MBON-γ5β′2a, as previously shown in other MBONs (Cohn et al., 2015; Hige et al., 2015; Berry et al., 2018; Handler et al., 2019).

### Compartment-specific depression of odor-evoked neurotransmitter release by KCs following odor-DAN pairing

Having established the condition for plasticity induction in MBON-γ5β′2a, we next applied the same odor-DAN pairing protocol to a brain expressing an acetylcholine (ACh) sensor (ACh3.0; Jing et al., 2020) in KCs (Fig. 2*A*), which enabled a rigorous comparison between the possible presynaptic and postsynaptic changes induced by this DAN-induced plasticity. Since MBON-γ5β′2a receives input from the γ5 and β′2 compartments, ACh release was recorded from the distal tip of the horizontal lobe of the MB. The imaging field of view included the γ4, γ5, and β′2 compartments, the identities of which were confirmed by the presence of tdTomato-tagged Chrimson within the γ5 and β′2 compartments and the absence of such label in the γ4 compartment. The γ4 compartment served as an internal control since it was illuminated by LED light, but its resident DAN axons were devoid of opsins to be activated because of selective Chrimson expression driven by the LexA line.

10.1523/ENEURO.0275-23.2023.f2-1 Extended Data Figure 2-1 Typical field of view during imaging experiment to show ROI selection guided by GFP-based sensor and tdTomato-tagged opsin signals. ***A***, ACh3.0 signal pseudocolored in green. Boundaries of ROIs corresponding to γ4, γ5, and β′2 marked in orange. Scale bar, 5 μm. ***B***, Chrimson88:tdTomato signal pseudocolored in magenta. Note that we intentionally drew ROI boundaries conservatively to avoid contamination of signals between compartments. Download Figure 2-1, EPS file.

10.1523/ENEURO.0275-23.2023.f5-1 Extended Data Figure 5-1 Response profiles of responsive γ5 boutons undergoing plasticity lack predictive features in their temporal patterns. ***A***, Rescaled prepairing responses to paired odor in boutons that will undergo changes of at least 20% after pairing, either via depression (*n* = 52; blue line) or potentiation (*n* = 51; orange line). Reproduced and modified from Figure 5*J*. ***B***, Same as ***A*** but for the odor-only condition (will be depressed, *n* = 37; will be potentiated, *n* = 66). Reproduced and modified from Figure 5*K*. ***C***, Mean rescaled value during early phase of prepairing odor responses. Repeated-measures two-way ANOVA to compare effect of pairing across odors (interaction *p *=* *0.8913). ***D***, Same as ***C*** but for middle phase. Repeated-measures two-way ANOVA to compare effect of pairing across odors (interaction *p *=* *0.7855). ***E***, Same as ***C*** but for late phase. Repeated-measures two-way ANOVA to compare effect of pairing across odors (interaction *p *=* *0.1069). ***F***, Principal component analysis of all paired odor-responsive γ5 boutons; mean prepairing odor responses segmented into 1-s bins; plasticity rates <20% (unfilled circles), depression ≥20% (filled blue), potentiation ≥20% (filled orange). Download Figure 5-1, EPS file.

ACh release was detected in all three compartments in response to odor presentation before pairing (Fig. 2*B–D*, right, black lines). After pairing, the γ5 compartment showed odor-specific plasticity (Fig. 2*B*, left) similar to that observed at the MBON-γ5β′2a dendrite (Fig. 1*E*). This took the form of post-pairing depression of ACh release in response to Odor A but not Odor B. Following reversal pairing, ACh release in response to Odor B was depressed while the response to Odor A was unchanged. This lack of Odor A recovery could be explained by the low signal-to-noise ratio because of the relatively small size of ACh sensor signals, but it may also support the contribution of postsynaptic mechanisms, as recently reported in the M4/6 group of MBONs (which includes MBON-γ5β′2a) in the context of intermediate-term memory (Pribbenow et al., 2022). In contrast to the odor-specific depression of the γ5 compartment, ACh release from the β′2 compartment was unaffected by pairing (Fig. 2*C*). The absence of plasticity in the β′2 compartment might mean that our odor-DAN pairing protocol did not efficiently engage plasticity in this particular compartment, either because of a different training threshold in the compartment or because of incomplete labeling of the corresponding DAN axons by the LexA driver. We also note that ACh sensor signals were analyzed in the entire β′2 compartment, which might have diluted the potential changes caused by β′2a-specific plasticity. Nevertheless, the robust depression observed at the MBON-γ5β′2a dendrite when only the γ5 compartment shows significantly depressed ACh release can be explained by the fact that this MBON receives the majority (∼77%) of its input from KCs in the γ5 compartment (Otto et al., 2020). Importantly, ACh release in the γ4 compartment, which serves as the internal control, was unchanged throughout the experiment (Fig. 2*D*). This compartment-specific and odor-specific depression of ACh release demonstrates the presynaptic nature of LTD, although the results do not rule out the possibility of coexisting postsynaptic mechanisms (Pribbenow et al., 2022).

### Odor-evoked bulk Ca^2+^ activity of the KC axon bundles does not mirror depression of neurotransmitter release

Since previous studies reported a paradoxical increase in odor-evoked Ca^2+^ activity in the KC presynaptic terminals after conditioning using natural reward or punishment (Yu et al., 2006; Wang et al., 2008; Akalal et al., 2010; Boto et al., 2014, 2019), we next asked whether LTD induced by odor-DAN pairing accompanies any changes in presynaptic Ca^2+^ activity. To test this, we used the same pan-KC driver from the ACh imaging experiments to drive expression of the Ca^2+^ sensor instead of the ACh sensor (Fig. 3*A*). We found that bulk Ca^2+^ activity in the MB lobes was not reflective of odor-DAN pairing-induced changes to KC ACh release or MBON Ca^2+^ activity. The γ5 compartment showed no change after pairing or reversal (Fig. 3*B*), in opposition to the depression of ACh release described above. The β′2 compartment, which showed unchanged ACh release, also remained unchanged by odor-DAN pairing (Fig. 3*C*). The control γ4 compartment was also unaffected (Fig. 3*D*). These findings outline a picture of DAN-induced plasticity, at least in the γ5β′2a compartments, in which LTD of the KC-to-MBON synapse exhibits itself as reduced activation of the MBON via reduced release of ACh by the KC axons wherein bulk Ca^2+^ activity is apparently spared from dopamine’s plastic effects.

### Odor-DAN pairing depresses MBON-γ1pedc Ca^2+^ activity and upstream KC ACh release while leaving KC Ca^2+^ activity unaffected

To determine whether our findings thus far made in the γ5β′2a compartments are generalizable to other MB compartments, we next focused on the γ1pedc compartments, as they represent aversive memory compartments (Aso et al., 2010) innervated by the protocerebral posterior lateral 1 (PPL1) DAN cluster, the other major subpopulation of DANs that innervate the MB lobes. We applied the same step-by-step strategy to MBON-γ1pedc that we applied to MBON-γ5β′2a: moving from Ca^2+^ activity at the MBON dendrites to ACh release from KC axons to bulk Ca^2+^ activity of KC axons (Fig. 4*A*).

As at the MBON-γ5β′2a dendrites, the MBON-γ1pedc dendrites displayed odor-specific depression after pairing followed by potentiation of the Odor A response and depression of Odor B response after reversal (Fig. 4*B*). Similarly, ACh release from the γ1 compartment and the subregion of the peduncle innervated by PPL1-γ1pedc DANs (the “γ1pedc compartments”) was depressed with odor specificity after pairing and reversal (Fig. 4*C*). The lack of recovery in Odor A-evoked ACh release after reversal was also consistent in the γ1pedc compartments. These effects were compartment specific, as the internal control, the neighboring γ2 compartment, showed no effect of odor-DAN pairing (Fig. 4*D*). Finally, bulk Ca^2+^ responses again showed no effect of odor-DAN pairing in either the γ1pedc (Fig. 4*E*) or γ2 compartments (Fig. 4*F*). Taken together, we conclude that LTD at KC-to-MBON synapses induced by odor-DAN pairing does not involve corresponding decrease in the bulk Ca^2+^ signals in the KC presynaptic terminals.

### Odor-evoked Ca^2+^ activity in individual presynaptic boutons shows heterogeneous changes after odor-DAN pairing

Although changes in the bulk Ca^2+^ activity of the KC axon bundles are often interpreted as one of the important indicators of MB plasticity, the absence of such changes demonstrated here does not necessarily mean that presynaptic plasticity does not involve Ca^2+^ changes in KCs. A recent study demonstrated that odor-shock pairing induces highly heterogeneous changes in Ca^2+^ activity of individual KC presynaptic boutons (Bilz et al., 2020). Therefore, what appears as stability in bulk Ca^2+^ activity may actually be a mix of potentiated and depressed synapses whose changes become masked when summed across the population.

To explore this possibility, we performed presynaptic Ca^2+^ imaging of individual KC boutons. To distinguish between signals from individual boutons, we expressed jGCaMP7f (Dana et al., 2019) in a sparse population of KCs using the stochastic expression system, SPARC (Isaacman-Beck et al., 2020), combined with the same DAN labeling strategies used before (Fig. 5*A*,*B*). Given the sparseness of odor representation by KCs, consistently finding axons that happen to express the Ca^2+^ sensor and also happen to respond to both MCH and OCT was not a reasonable expectation. To address this limitation, we presented up to eight odors to the fly at the beginning of each experiment to find an odor to which the Ca^2+^ sensor-expressing axons were responsive. In the event that a response-evoking odor was identified, it was assigned as Odor A and a second odor, which did not evoke any recognizable response, was selected randomly from the panel as Odor B. The experiment then followed the same odor-DAN pairing protocol as described before (Fig. 1*B*) except that the “Reversal” and “Post-reversal” steps were omitted because Odor B did not typically evoke responses in the labeled KCs. Half of the flies were assigned to the “paired” group, in which the “Pairing” step was completed normally, and the other half were assigned to the “odor-only” group, in which the “Pairing” step was completed with an unpowered LED. Since the γ5 compartment was the only compartment that displayed presynaptic plasticity following odor-DAN pairing, we focused our bouton imaging effort there, with the γ4 compartment continuing as an internal control.

After odor-DAN pairing, KC axonal boutons in the γ5 compartment (“γ5 boutons”) exhibited highly heterogenous changes (Fig. 5*C*). Because of this heterogeneity, indiscriminate pooling of all γ5 boutons together did not reveal overt effects of odor-DAN pairing (Fig. 5*C*), except a slight potentiation detected in the odor-only boutons. But the results were reminiscent of our preceding bulk Ca^2+^ imaging experiments in that changes in Ca^2+^ dynamics did not reflect our observed changes in ACh release. Neither group of KC axonal boutons in the γ4 compartment showed an effect of pairing (Fig. 5*D*). However, the clear heterogeneity in size and temporal dynamics of bouton responses (e.g., paired γ5 bouton responses ranging from −3.43 to 22.61 in *z* score) led us to wonder whether mass bouton comparisons could be delegitimized by inadvertent combination of distinct response types. Therefore, we applied a divisive filter to distinguish response types from among the population (Fig. 5*E–H*, filter demonstrated using pre-pairing responses from all γ5 boutons from paired and odor-only groups). Boutons in which the 10-s period following odor onset displayed a mean *z* score greater than a *p *=* *0.05 threshold (*z* score of 1.6449) were defined as “Positive” (Fig. 5*E*). This conservative definition minimized the inclusion of any false positives within the group. Remaining boutons were defined as “Negative” if the mean *z* score of any 1-s postbaseline period surpassed a negative *p *=* *0.05 threshold (*z* score of −1.6449; Fig. 5*F*) or as “Biphasic” if a bouton showed mean *z* scores of separate 1-s postbaseline periods that surpassed both the positive and negative thresholds (Fig. 5*G*). After applying these conservative criteria, remaining uncategorized boutons were defined as “Unresponsive” (Fig. 5*H*). Since “Negative” and “Biphasic” categories represented only a small fraction of boutons and therefore lacked sufficient power for in-depth study, our subsequent analysis focused on the “Positive” category (referred to as “positive responder boutons”). By itself, this group still represented striking diversity in response time course (Fig. 5*I*) as well as in the changes after pairing (i.e., 111 and 140 of a total 251 positive responder γ5 boutons depressed and potentiated, respectively).

### Temporal characteristics of pre-pairing odor response profiles do not predict the sign of post-pairing plasticity

There is evidence that the temporal alignment of odor presentation and DAN activity influences the sign of evoked plasticity at the MBON dendrite (Handler et al., 2019) wherein odor preceding DAN activation promotes depression while DAN activation preceding odor promotes potentiation. Combined with the observation that bouton response profiles are diverse (Fig. 5*I*) and the fact that subsets of KCs show activity peaks at different points within the odor presentation window (Vrontou et al., 2021), we first asked whether some temporal response profile features of a given bouton could predict the direction of plasticity evoked by odor-DAN pairing. To this end, we first expanded the positive responder bouton group to include boutons whose pre-pairing or post-pairing odor response satisfied the established criteria. This redefinition is critical because including boutons that qualify as “Positive” only at pre-pairing could introduce selection bias that artificially constrains our dataset’s ability to detect anything besides depression. Results of comparison of raw response profiles would be distorted by the wide variety in response magnitude and shape (Fig. 5*I*). Therefore, to extract the temporal characteristics of the positive responder boutons, each bouton’s pre-pairing odor response was rescaled to a minimum value of 0 and a maximum value of 1. The boutons were then divided into two groups: the group that “will be depressed” by odor-DAN pairing included boutons whose odor response decreased by ≥20% after odor-DAN pairing (Fig. 5*J*,*K*, blue lines), and the group that “will be potentiated” included boutons whose odor response increased by ≥20% after odor-DAN pairing (Fig. 5*J*,*K*, orange lines). We found no obvious superficial differences between the rescaled response profiles of these plasticity groups for the paired (Fig. 5*J*) or the odor-only γ5 boutons (Fig. 5*K*). Breaking down the response profiles into early, middle, and late phases did not reveal differences between the plasticity groups (Extended Data Fig. 5-1*A–E*) and increasing the plasticity threshold to ≥40% delivered the same conclusion (data not shown). Furthermore, principal component analysis applied to the rescaled response profiles divided with greater temporal resolution (response profiles segmented into 1 s bins) did not reveal obvious clustering of the plasticity groups in the principal component space (Extended Data Fig. 5-1*F*). Based on the agreement between these two strategies, we conclude that temporal characteristics of pre-pairing odor response profiles alone cannot be a predictor of the eventual plasticity displayed by a bouton after odor-DAN pairing.

### Magnitude of naive odor response biases the direction of post-pairing plasticity

Since temporal characteristics of response profiles did not explain the observed plasticity, we next examined the relationship between response magnitudes and plasticity outcomes. We started by plotting the mean *z* score from the pre-pairing odor response against that of the post-pairing odor response for each positive responder bouton in each compartment. Again, we included the boutons that showed significant responses at *either* pre-pairing or post-pairing to remedy the bias concerns mentioned above. Qualitatively, it seemed that the paired γ5 boutons with the strongest pre-pairing, or naive, responses appeared most often below the line of unity, suggesting depression, while the corresponding subset of the odor-only γ5 boutons appeared scattered around it (Fig. 6*A*). Conversely, the paired γ5 boutons with the weakest naive responses tended to rise above the line of unity, suggesting potentiation, while the odor-only γ5 boutons appeared more balanced. Linear regression analysis and slope comparisons supported this notion. In contrast, neither experimental group of γ4 boutons showed an inclination in one direction or the other (Fig. 6*B*).

10.1523/ENEURO.0275-23.2023.f6-1 Extended Data Figure 6-1 Comparing paired odor responsive boutons from experimental flies against paired responsive boutons from genetic control (G.C.) flies. ***A***, Prepairing mean *z* score plotted against postpairing mean *z* score for all γ5 boutons responsive to odor. Orange points represent paired odor responsive boutons from experimental flies (*n* = 116 boutons, *N* = 10 flies); black points represent paired odor responsive boutons from G.C. flies (*n* = 129, *N* = 11); solid lines represent linear regression lines compared by ANCOVA (*p *=* *0.3005); hashed black line marks line of identity. ***B***, Same as ***A*** but for odor responsive boutons in γ4 compartment (paired *n* = 84, *N* = 11; G.C. *n* = 116, *N* = 11); linear regression lines compared by ANCOVA (*p *=* *0.1438). ***C***, Minimum-to-maximum box plots of mean total *z* score of “strong responder” boutons within γ5 compartment (paired *n* = 71, G.C. *n* = 93). Fine lines indicate data from individual flies; bold lines indicate mean; horizontal bar within box indicates median. Repeated-measures two-way ANOVA to compare effect of pairing across odors (interaction *p *=* *0.0469) and within odors at each stage of the experiment (multiple comparisons from left to right: *p *=* *0.0016, 0.6231). Lower, Left, Paired odor mean response profile (±SEM, shaded area) at prepairing (black), postpairing (red), and postreversal (blue). Lower, Right, Same as left but for paired odor in G.C., flies. ***D***, Same as ***C*** but for “strong responder” boutons in γ4 compartment (paired *n* = 61, G.C. *n* = 78). Repeated-measures two-way ANOVA to compare effect of pairing across odors (interaction *p *=* *0.3248). ***E***, Same as ***C*** but for “weak responder” boutons in γ5 compartment (paired *n* = 45, G.C. *n* = 36). Repeated-measures two-way ANOVA to compare effect of pairing across odors (interaction *p *=* *0.0051) and within odors at each stage of the experiment (multiple comparisons from left to right: *p *<* *0.0001, 0.0397). ***F***, Same as ***C*** but for “weak responder” boutons in γ4 compartment (paired *n* = 23, G.C. *n* = 38). Repeated-measures two-way ANOVA to compare effect of pairing across odors (interaction *p *=* *0.3309). Download Figure 6-1, EPS file.

For further dissection of these tendencies, we subcategorized the positive responders into “strong” and “weak” responders using the threshold of *p *=* *0.005 (*z* score of 2.576). Strong responder boutons in the γ5 compartment showed depressed Ca^2+^ responses under the paired condition while those of the odor-only condition showed no change (Fig. 6*C*). In contrast, weak responder boutons of the γ5 compartment in the paired condition showed greater potentiation than those in the odor-only condition (Fig. 6*E*). In the neighboring γ4 compartment, both groups of strong responder boutons remained unchanged (Fig. 6*D*), and the effect on weak responder boutons was again equivalent (Fig. 6*F*). To confirm these findings, we also performed parallel experiments using genetic control flies, which lacked the LexA driver for Chrimson. The differences within and across compartments as well as the similarities within the γ4 compartment held up when comparing the paired boutons with the genetic control rather than with the odor-only group (Extended Data Fig. 6-1). These results indicate that odor-DAN pairing evokes bidirectional plasticity of Ca^2+^ responses of individual KC boutons and that the sign of this plasticity, at least in part, depends on the magnitude of the bouton’s naive odor response.

### Sibling boutons of the same axon undergo distributed and divergent plasticity

Although our results indicate that boutons with strong naive odor responses are more likely to undergo depression, we still observed considerable heterogeneity in the direction of plasticity within that group of strong responders. We therefore attempted to identify a group of boutons that exhibit more uniform plasticity. We reasoned that boutons belonging to spiking KCs are most likely to undergo depression and that most, if not all, boutons on spiking axons should show positive naive odor responses. Testing this prediction required assigning boutons to their parent axons. While the proportion of KCs expressing jGCaMP7f was consistent across flies, the arrangement of those axons within the MB lobe was expectedly variable, with some flies showing easily trackable axons and others with heavy overlap that precluded clean tracing. So, we identified every possible set of sibling boutons (from both the γ4 and γ5 compartments) sharing a parent axon and then included in our subanalysis any parent axon housing at least eight boutons. With this strategy, we were able to assign 419 boutons (52% of γ4 and γ5 boutons in our dataset) to 24 parent axons. To identify axons belonging to likely spiking KCs, we set a constraint that at least 80% of the sibling boutons must respond positively to odor presentation (Fig. 7*A*,*B*, left of the vertical hashed line). All other axons are classified as “likely nonspiking” (Fig. 7*A*,*B*, right of the vertical hashed line).

We found that across both paired and odor-only groups, likely spiking axons are populated almost entirely by strong responder boutons: of 111 γ5 boutons residing on likely spiking axons, 97 naïvely responded strongly to odor. This result supports our idea that axons with high bouton response rates are likely spiking axons, as we expect that response magnitude is greater in a spiking axon than a nonspiking axon. It also suggests that weak bouton responses on the axons with low response rates may be activated by local excitation in the MB lobe. However, the boutons of likely spiking axons are not uniform in their plasticity; those of the paired group did not exclusively undergo depression (Fig. 7*A*, axon #1 compared with axons #2–4). In the axons that we could survey, this diverse expression of plasticity by sibling boutons along the parent axon did not appear segregated into plasticity-specific spatial domains. Instead, potentiated and depressed boutons appeared randomly distributed along the neurite in both the paired (Fig. 7*C–E*) and odor-only (Fig. 7*F*) conditions. The result that not all boutons of spiking KCs automatically undergo depression following odor-DAN pairing suggests that coincidence of KC activation and dopaminergic input may not be the sole determinants of synaptic plasticity.

## Discussion

This study describes the effects of DAN-induced plasticity on the presynaptic side of the KC-to-MBON synapse using odor-DAN pairing via optogenetic control of DANs. This strategy enabled us to systematically compare activity at key sites along the KC-to-MBON transmission pathway under identical conditions while focusing on the effect of dopamine. Using an odor-DAN pairing protocol verified to induce suppression of the odor response at the MBON dendrites, we observed a corresponding compartment-specific depression of odor-evoked neurotransmitter release alongside unchanged odor-evoked Ca^2+^ activity of the KC axon bundles in both the MBON-γ5β′2a and MBON-γ1pedc circuits. Subsequent imaging with single bouton resolution revealed an underlying mixture of magnitude-driven bidirectional plasticity in odor-evoked Ca^2+^ activity. Our results provide a new reading on seemingly inconsistent reports on the effect of learning-related plasticity on presynaptic Ca^2+^ activity, and they challenge the field’s current view of the cooperative relationship between presynaptic Ca^2+^ influx and dopaminergic input.

There has been evidence for a full range of effects of learning-related plasticity on odor-evoked Ca^2+^ activity of the KC axon bundles: depressed (Zhang et al., 2019), potentiated (Yu et al., 2006; Wang et al., 2008; Akalal et al., 2010; Boto et al., 2014, 2019; Louis et al., 2018), and unchanged (Louis et al., 2018). While these studies are essential for describing the circuit-level changes in this process and for describing processes underlying natural learning, it has been difficult to tell which of these observed phenomena are the direct outcome of the synaptic plasticity induced by DAN activation. Therefore, the present study’s effort to isolate the contribution of DANs adds important context to this body of work. This missing context is significant because, although it is well-established that the behavior-tuning output of the MB is depressed by dopamine-dependent plasticity of the KC-to-MBON synapse (Séjourné et al., 2011; Cohn et al., 2015; Hige et al., 2015; Owald et al., 2015; Berry et al., 2018; Handler et al., 2019), many studies looking for corresponding presynaptic changes found conflicting results. For example, post-training depression is well-established in MBON-γ5β′2a (Owald et al., 2015; Handler et al., 2019; Pribbenow et al., 2022), yet post-training Ca^2+^ responses in KC axon bundles are seemingly potentiated (Boto et al., 2014; Louis et al., 2018). Similarly, while MBON-γ1pedc shows post-training depression (Felsenberg et al., 2018; Hige et al., 2015; Perisse et al., 2016), potentiation (Wang et al., 2008; Boto et al., 2014) or no change (Louis et al., 2018) in Ca^2+^ responses of KC axon bundles are reported. On the other hand, complementary studies using recently developed ACh sensors are in agreement with MBON depression (Zhang et al., 2019; Stahl et al., 2022; Noyes and Davis, 2023; Zeng et al., 2023). One explanation for the ostensible inconsistency among reports is minor differences in experimental design or genetic tools. But the results of the present study offer an alternative interpretation. Our result that odor-DAN pairing does not induce changes to bulk presynaptic Ca^2+^ odor responses (while still causing depression of ACh release and MBON activity) suggests that the previously observed changes in presynaptic Ca^2+^ activity may not be dopamine-dependent or even directly related to immediate changes in ACh release. This does not mean that bulk changes in Ca^2+^ are not learning-related. Instead, it may simply be a product of a different neuromodulator (Zeng et al., 2023), one of the circuit-wide effects induced by conditioning (Perisse et al., 2016; Felsenberg et al., 2017, 2018; McCurdy et al., 2021; Villar et al., 2022; Yamada et al., 2023), or even reflective of processes involved in intermediate or long-term memory (Yu et al., 2006; Wang et al., 2008; Akalal et al., 2010; Turrel et al., 2022) rather than immediate memory, which is most relevant to our experimental condition.

How do the apparently balanced but opposing depression and potentiation of presynaptic Ca^2+^ responses give rise to a decisively depressed odor response in the MBON? First, our experiments most likely underestimate the presynaptic Ca^2+^ plasticity because the Ca^2+^ sensor may not capture all Ca^2+^ signals related to neurotransmitter release. Second, dopamine-induced LTD might additionally involve modulation of presynaptic factors that reside downstream of Ca^2+^ influx through voltage-gated Ca^2+^ channels, such as vesicular release machinery. Finally, it is possible that strongly responding boutons, which exhibited depression after pairing, are the ones that mainly contribute to synaptic transmission. On the other hand, weakly responsive boutons may represent so-called “deaf” synapses (Voronin and Cherubini, 2004) that are recovering from postsynaptic changes in receptor arrangement (Pribbenow et al., 2022) induced by previous experiences. Testing these possibilities would require single-bouton imaging of ACh release, which, to our knowledge, is not feasible with currently available sensors.

The observation of depression of strongly responsive boutons dovetails with the field’s current understanding, as demonstrated by the temporal sensitivity of plasticity induction: DAN activation that coincides with or follows shortly after odor presentation (“forward” pairing) leads to depression (Séjourné et al., 2011; Cohn et al., 2015; Hige et al., 2015; Owald et al., 2015; Handler et al., 2019). However, to our knowledge, our observation of potentiation of weakly responsive boutons does not fit with any previously described potentiation in the MB. Previously reported potentiation relied on dopaminergic input and the lack of odor-evoked Ca^2+^ influx, via either DAN activation preceding odor presentation (“backward” pairing; Berry et al., 2018; Handler et al., 2019) or DAN activation alone (Cohn et al., 2015). Neither of these explanations are likely to apply to the potentiated described here, based on our analysis of temporal dynamics (Fig. 5*J*,*K*; Extended Data Fig. 5-1) and our strict threshold for identifying significantly responsive boutons at pre-pairing (Fig. 5*E–H*). Our results call for experimental tests for further refinement of the plasticity rule; although, finely manipulating the presynaptic Ca^2+^ influx to mimic the weakly responsive boutons might be difficult.

Our study uncovered remarkable heterogeneity across boutons in both naive responses and evoked plasticity even among those residing on the same, likely spiking axons. One likely source of this heterogeneity is local modulatory inputs at the axons. KCs make many axoaxonic connections with other targets (Takemura et al., 2017). This includes reciprocal connections with other KCs, with DANs (Cervantes-Sandoval et al., 2017), with an inhibitory interneuron called the anterior paired lateral (APL) neuron (Amin et al., 2020), and with the serotoninergic dorsal paired medial (DPM) neuron. KCs also make gap junctions with other KCs (Q. Liu et al., 2016). Notably, a recent study showed that muscarinic type-B receptors that preferentially localize at KC axons mediate lateral inhibition of both odor-evoked Ca^2+^ activity and dopamine-evoked cAMP production in neighboring axons (Manoim et al., 2022). Based on this, weakly or strongly responsive boutons that did not undergo depression may have been under the influence of local modulatory inputs. Additionally, innervation by DANs is not uniform in the microscopic view; a connectome study revealed that only 6% of total KC-MBON synapses have a DAN terminal within 300 nm (Takemura et al., 2017). Moreover, PAM DANs in the same compartment are functionally heterogeneous (Otto et al., 2020). Thus, the observed heterogeneity of plasticity across boutons might reflect relative proximity to the nearest DAN terminal and the identity of that DAN. Another related possibility is that the origin of the observed Ca^2+^ response differs in weakly responsive boutons, which may lead to distinct outcomes of plasticity; backward, but not forward, odor-DAN pairing induces efflux of Ca^2+^ from the endoplasmic reticulum, leading to potentiation of synapses (Handler et al., 2019). It is also possible that the response magnitude and plasticity rule varies depending on the postsynaptic partner; although KC presynaptic boutons facing non-MBON targets are often shared with MBONs as well.

Our finding of bidirectional plasticity and heterogeneity of naive and post-training odor-evoked Ca^2+^ activity is largely in agreement with a recent study, which, to our knowledge, is the only other study to examine plasticity in KC axonal boutons (Bilz et al., 2020). It further revealed that their odor-shock pairing-induced bidirectional plasticity led to decorrelation within boutons of the same axon and across axons of the same compartment, enabling an “expansion of coding space” available to each KC. While the single axon analysis here is too limited to support this conclusion directly, the mode of plasticity we describe is consistent with their results and builds on their work by identifying the magnitude-driven bias underlying our common observation of bidirectional plasticity. However, it is still an open question how dopamine may shape the odor representation signal, whether it be compartment-wide, as offered by Bilz et al. (2020), or synapse-by-synapse, as proposed here. The same question is waiting to be addressed for other neuromodulators as well, such as DPM-derived serotonin that may extend dopamine’s functional plasticity window within the KC axon (Zeng et al., 2023). Such investigation will also be of value for future modeling studies, which to this point must assume simple spiking of KCs and equal or random weights to KC-to-MBON synapses (Smith et al., 2008; Huerta and Nowotny, 2009; Peng and Chittka, 2017; Abdelrahman et al., 2021; Hafez et al., 2023). The intersection of these approaches will be especially important for understanding the subcellular mechanisms of learning-related processes, particularly as they appear to be distributed across functional units, whether they be compartmentalized axon bundles or boutons along an individual axon.

## Synthesis

Reviewing Editor: Isabel Perez-Otaño, Instituto de Neurociencias Decisions are customarily a result of the Reviewing Editor and the peer reviewers coming together and discussing their recommendations until a consensus is reached. When revisions are invited, a fact-based synthesis statement explaining their decision and outlining what is needed to prepare a revision will be listed below. The following reviewer(s) agreed to reveal their identity: Karla Kaun.

Both reviewers found merit in the work, stating that it advances our understanding of plasticity rules encoding associative learning within the Drosophila mushroom body. They had some questions/concerns that I hope you can address in a revised manuscript.

## Author Response

Both reviewers found merit in the work, stating that it advances our understanding of plasticity rules encoding associative learning within the Drosophila mushroom body. They had some questions/concerns that I hope you can address in a revised manuscript.

Reviewers´comments:

Reviewer#1 Davidson et al is an elegant study that uses the Drosophila mushroom body system to address how coincidence of odor plus dopaminergic input that phenocopies punishment influences synaptic plasticity in pre- and postsynaptic sites. The authors determine whether synaptic plasticity as a consequence of odor-shock memory is accompanied by a corresponding decrease in odor evoked calcium activity in the Kenyon cell presynaptic terminals. The theory in the field is that coincidence of presynaptic calcium and dopaminergic input triggers depression. Their results suggest that the plasticity outcome is more heterologous than expected, and that plasticity outcome is finely controlled by the activity of individual synapses and local neuromodulatory signals. This is a key contribution to the field as it highlights complexity of the physiology of learned responses at multiple scales. Overall this is a rigorous study that supports the main conclusions as written. The methods and results were clear, and topics discussed were appropriate to the findings of the study. No major concerns were noted in the study, but addressing several relatively minor concerns would significantly increase rigor and reproducibility of the study. 1) From the study, it is unclear whether the authors are studying male or female flies. Please clearly write the sex of animals used in methods and results. If both males and females were used, please perform the relevant test for sex differences and write this into the results.

We used only females for this study. We clarified this point by adjusting the language in the methods section as follows: “At 1-2 days post-eclosion, flies for experiments were selected based on sex and genotype after sorting under brief CO2 anesthesia. All experiments used female flies that fed on standard food supplemented with all-trans-retinal...” (lines 116- 118)

2) Please provide an example of a MB image that outlines the MB compartments as the authors classified the compartments in the methods or supplemental material.

Figure 2-1 (legend at lines 823-827) has been added to show ROI selection during bulk ACh and Ca2+ imaging experiments. An additional statement has been added to the Methods section (lines 198-199) and the figure legend (lines 826-827) to explain that ROI boundaries were intentionally drawn within actual compartmental boundaries to avoid cross compartmental “contamination,” based on Cohn et al., 2015.

3) Please provide raw data in supplement so scientists trying to reproduce work would be able to compare the sensitivity of the sensors used (6f, 6s, 7f and ACh sensor).

The requested raw data are a large quantity of imaging data, so it is not practical to post them as supplementary file. We instead added the following statement: “All data generated in this work will be shared upon reasonable request to the corresponding author.” (line 233)

4) In results for Figure one, please indicate the rationale for the types of stimulations used in the results. Please also indicate whether 5 pairings of 1ms x 4@2Hz stimulations are sufficient to induce behavior, and 5 stims with non-paired odor is sufficient to reverse these effects.

We added the following statement for justification and clarification: “We used odor-DAN pairing protocols similar to those used in behavioral experiments that activated the same or similar DAN subpopulations (Aso and Rubin, 2016; Yamada et al., 2023). Although we did not test whether our exact training protocols can induce behavioral changes, they successfully induced changes in olfactory responses in the MBONs that are consistent with previously reported learning-related changes.” (lines 182-186)

5) In the discussion for Figure 2, please discuss the unusual reversal results in 2B. Please also speculate why there is an odor-specific effect in 2B in the discussion.

In the original manuscript, we already discussed the absence of reversal pairing-induced plasticity in ACh3.0 signal, which reads, “This lack of Odor A recovery could be explained by the low signal-to-noise ratio due to the relatively small size of ACh sensor signals, but it may also support the contribution of postsynaptic mechanisms, as recently reported in the M4/6 group of MBONs (which includes MBON-γ5β′2a) in the context of intermediate-term memory (Pribbenow et al., 2022).” (lines 285-288). We feel there are no additional points to discuss regarding this matter. Odor-specific effect in 2B is as normally observed in any other plasticity we presented in this manuscript; pairing only affects the response of the paired odor.

6) In Fig 3 the y4 and y5 responses look really similar - is it possible that the authors accidently mislabelled the compartments or the compartment labelling was slightly inaccurate which could result in doubling up of data? Providing screenshots with outlines of how the different lobes were characterized would be helpful to readers.

The responses do look similar, but we confirmed that there is no mislabeling or inaccuracy in compartment labeling. In our experimental design, every imaged fly that contributes a g4 compartment also contributes a g5 compartment, and the same KC axons run through those compartments, so there should be a strong correlation between the activities measured at each compartment (known as noise correlation). In response to the comment above, images with ROI boundaries are now included in Figure 2-1. Also, an additional statement has been added to the Methods section (lines 198-199) and the figure legend (lines 825-826) to explain that ROI boundaries were intentionally drawn within actual compartmental boundaries to avoid cross-compartmental “contamination,” based on Cohn et al., 2015.

7) It is possible that one of the reasons the Ach response doesn’t match Ca2+ is different sensitivity of the sensors. What if tried more sensitive sensor like GCaMP8?

We think it is possible that results are different if we use more sensitive calcium sensors. However, in our hands, ACh3.0 has a lower signal-to-noise ratio in odor responses compared to any GCaMPs we used. This is why we discussed as such in the point mentioned in Fig. 2B.

8) Figure 5 provides the most important and elegant experiments in this manuscript. Thus, it is necessary to include all elements demonstrating the rigor of this experiment. Please explain the switch from GCaMP6 to GCaMP7f for these experiments. Please indicate N’s of flies (as well as boutons / fly) for datasets. Consider adding additional measures that might explain heterogeneity such as morphology (size, shape), baselines activity prior to odor-responses, etc. In the discussion, please also discuss the data in 5H as there were a number of traces where there is an increase post-odor, but it’s unclear why that trace would not fit into a biphasic or positive response. We added a brief statement to explain why we used jGCaMP7f for bouton imaging, which reads, “We used jGCaMP7f instead of GCaMP6f for these experiments because jGCaMP7f gave a higher basal fluorescence intensity in our preliminary experiments, which was advantageous for imaging small structures.” (lines 157-159). As for other potential factors that might explain heterogeneity of bouton plasticity, we feel we exhausted parameters that are reliably analyzable in our current dataset. In particular, the power of our analysis was limited by the number of strongly responsive axons. We added the number of flies used for this dataset in the figure legends for Figure 5 (lines 869 and 875) and Figure 6-1 (lines 926 and 927 and 929). We also adjusted the y-scale of the plot in Figure 5H as we believe that the difference in scale led to the impression that there are too many false negatives.

Reviewer#2 In the study titled “Nonlinear Relationship Between Presynaptic Calcium Influx and Dopamine Induced Long-Term Depression in the Drosophila Mushroom Body,” the authors delve into the intricate synaptic mechanisms underpinning associative learning within the Drosophila mushroom body (MB). By investigating the interaction between odor-evoked calcium influx and dopaminergic input, they employ optogenetic manipulation of dopaminergic neurons (DANs) to induce long-term depression (LTD) in KC’s presynaptic terminals through odor-DAN pairing. While previous research has established the suppression of odor response at MBON dendrites, this study presents a novel perspective by demonstrating compartment-specific depression of neurotransmitter release at KCs’ presynaptic terminals. Intriguingly, the study reveals that KC axon bundles’ overall odor-evoked calcium activity remains unaffected. Furthermore, the authors uncover heterogeneous bidirectional plasticity in calcium activity through high-resolution imaging at individual presynaptic boutons. These findings challenge the conventional understanding of the cooperative relationship between presynaptic calcium influx and dopaminergic input, underscoring the intricate nature of synaptic plasticity in associative learning. This study significantly advances our understanding of the intricate mechanisms governing associative learning within the Drosophila MB model system by integrating multifaceted approaches, from optogenetics to single-bouton imaging. The figures are precise, and the text is well-written and easy to follow.

Minor comments.

If analyzed in bulk, ca2+ changes at KC boutons are unaltered after odor-DAN pairing. However, when attention is paid to individual buttons, it is possible to observe that the responses are heterogeneous. However, when the authors use the ACh sensor, they can observe an apparent decrease in Acetylcholine release from KC axons. If analyzed at single button resolution, like with the Ca imaging, would the authors observe the same level of heterogeneity in ACh release? These could indicate that each bouton is receiving different DAN input or being modulated differentially by other neurons.

Imaging ACh release from a single bouton is one of the dream experiments we’d like to do as we mentioned in discussion, “Testing these possibilities would require single-bouton imaging of ACh release.” (lines 525-26). However, the current sensor is not sensitive enough. We added some words to clarify this point at the end of this sentence: “, which, to our knowledge, is not feasible with currently available sensors”. (line 526)

Concerning the previous comment, the authors indicate in the discussion that many different neuromodulators could be affecting the KC-MBON synapsis. Do these synapses are all innervated by DANs? Could this also be why the heterogeneity in Ca imaging response in KC axons is observed?

This is an important discussion point we forgot to mention. We now added the following passage: “Additionally, innervation by DANs is not uniform in the microscopic view; a connectome study revealed that only 6% of total KC-MBON synapses have a DAN terminal within 300 nm (Takemura et al., 2017). Moreover, PAM DANs in the same compartment are functionally heterogeneous (Otto et al., 2020). Thus, the observed heterogeneity of plasticity across boutons might reflect relative proximity to the nearest DAN terminal and the identity of that DAN.” (lines 550-554)
